# Bis(1-benzyl­piperazine-1,4-diium) hexa­chloridocadmate(II) dihydrate

**DOI:** 10.1107/S1600536810026073

**Published:** 2010-07-07

**Authors:** Meher El Glaoui, Matthias Zeller, Erwann Jeanneau, Cherif Ben Nasr

**Affiliations:** a Laboratoire de Chimie des Matériaux, Faculté des Sciences de Bizerte, 7021 Zarzouna, Tunisia; b Youngstown State University, Department of Chemistry, One University Plaza, Youngstow, Ohio 44555-3663, USA; c Universtié Lyon1, Centre de Diffractométrie Henri Longchambon, 43 Boulevard du 11 Novembre 1918, 69622 Villeurbanne Cedex, France

## Abstract

The asymmetric unit of the title compound, (C_11_H_18_N_2_)_2_[CdCl_6_]·2H_2_O, consists of one 1-benzyl­piperazine-1,4-diium dication, one water mol­ecule and one-half of a [CdCl_6_]^4−^ anion, located on an inversion centre. The crystal packing is governed by an extensive three-dimensional network of inter­molecular O—H⋯Cl, C—H⋯Cl, N—H⋯O and N—H⋯Cl hydrogen bonds, two of them bifurcated.

## Related literature

For *meta*-chlorido complexes, see: El Glaoui, Jeanneau, *et al.* (2009 ▶); El Glaoui, Kefi *et al.* (2009 ▶). For the role of C—H⋯Cl hydrogen bonds, see: Janiak & Scharmann (2003 ▶. For a discussion of Cd—Cl distances and Cl—Cd—Cl bond angles, see: Bala *et al.* (2006 ▶).
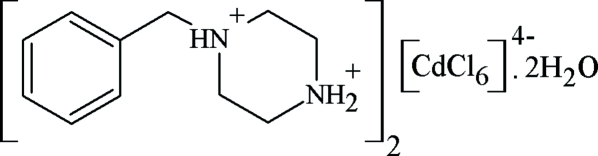


## Experimental

### 

#### Crystal data

(C_11_H_18_N_2_)_2_[CdCl_6_]·2H_2_O
*M* *_r_* = 717.68
Monoclinic, 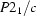

*a* = 12.734 (2) Å
*b* = 9.1686 (14) Å
*c* = 13.216 (2) Å
β = 103.249 (3)°
*V* = 1502.0 (4) Å^3^
*Z* = 2
Mo *K*α radiation
μ = 1.29 mm^−1^
*T* = 100 K
0.55 × 0.45 × 0.25 mm

#### Data collection

Bruker SMART APEX CCD diffractometer
Absorption correction: multi-scan (*SADABS*; Bruker, 2009 ▶) *T*_min_ = 0.622, *T*_max_ = 0.746
11244 measured reflections
4446 independent reflections
4123 reflections with *I* > 2σ(*I*)
*R*_int_ = 0.016

#### Refinement

*R*[*F* ^2^ > 2σ(*F* ^2^)] = 0.022
*wR*(*F* ^2^) = 0.057
*S* = 1.07
4446 reflections
172 parameters
3 restraints
H atoms treated by a mixture of independent and constrained refinement
Δρ_max_ = 0.51 e Å^−3^
Δρ_min_ = −0.83 e Å^−3^

### 

Data collection: *APEX2* (Bruker, 2009 ▶); cell refinement: *SAINT* (Bruker, 2009 ▶); data reduction: *SAINT*; program(s) used to solve structure: *SHELXTL* (Sheldrick, 2008 ▶); program(s) used to refine structure: *SHELXTL*; molecular graphics: *DIAMOND* (Brandenburg, 1998 ▶); software used to prepare material for publication: *SHELXTL*.

## Supplementary Material

Crystal structure: contains datablocks global, I. DOI: 10.1107/S1600536810026073/hb5541sup1.cif

Structure factors: contains datablocks I. DOI: 10.1107/S1600536810026073/hb5541Isup2.hkl

Additional supplementary materials:  crystallographic information; 3D view; checkCIF report

## Figures and Tables

**Table 1:** Hydrogen-bond geometry (Å, °)

*D*—H⋯*A*	*D*—H	H⋯*A*	*D*⋯*A*	*D*—H⋯*A*
N2—H2*A*⋯Cl1^i^	0.92	2.58	3.3383 (11)	140
N2—H2*A*⋯Cl2^i^	0.92	2.59	3.2672 (11)	131
N2—H2*B*⋯Cl2^ii^	0.92	2.47	3.1846 (11)	135
N2—H2*B*⋯Cl3^ii^	0.92	2.58	3.2799 (12)	133
N1—H1⋯O1	0.89 (1)	1.92 (1)	2.7945 (16)	170 (2)
O1—H1*A*⋯Cl1	0.84 (2)	2.39 (2)	3.1678 (11)	155 (2)
O1—H1*B*⋯Cl3^iii^	0.83 (2)	2.42 (2)	3.2152 (11)	161 (2)
C9—H9*A*⋯Cl3^ii^	0.99	2.83	3.331 (2)	112
C9—H9*B*⋯Cl3^iii^	0.99	2.85	3.659 (3)	139
C10—H10*A*⋯Cl3^iii^	0.99	2.73	3.565 (2)	143
C10—H10*B*⋯Cl2^ii^	0.99	2.84	3.340 (4)	112
C11—H11*A*⋯Cl1^ii^	0.99	2.71	3.626 (1)	154
C11—H11*B*⋯Cl1	0.99	2.72	3.587 (1)	146

Symmetry codes: (i) 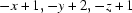; (ii) 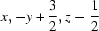; (iii) 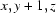.

**Fig. 1.**
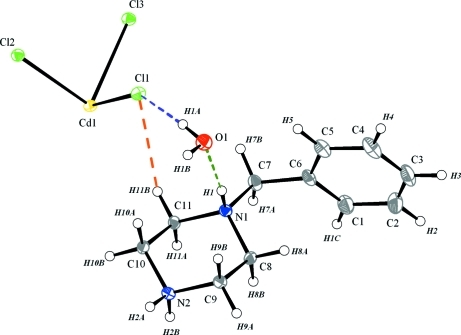
A view of the title compound, showing 50% probability displacement ellipsoids, arbitrary spheres for the H atoms, and the atom numbering scheme.

**Fig. 2.**
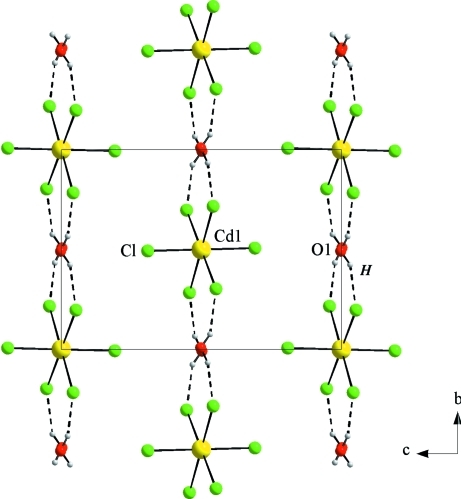
Projection along the a axis of the inorganic chains in (C11H18N2)2CdCl6.2H2O. Hydrogen bonds are denoted by dotted lines.

**Fig. 3.**
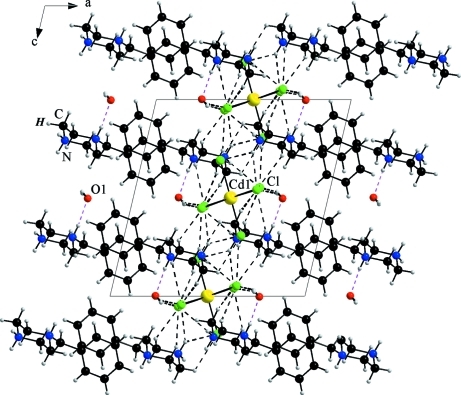
The packing of (C11H18N2)2CdCl6.2H2O, viewed down the b axis. Hydrogen bonds are denoted by dotted lines.

## supplementary crystallographic information

### Comment

As a part of our ongoing investigations in molecular salts containing *meta*-chlorido complexes (El Glaoui, Jeanneau, *et al.*, 2009; El Glaoui, Kefi *et al.*, 2009), we present here the crystal structure of one such compound, (C_11_H_18_N_2_)_2_CdCl_6_.2H_2_O, (Fig. 1). The asymmetric unit of its structure consists of one 1-benzylpiperazine-1,4-diium dication doubly protonated at the N1 and N2 nitrogen atoms, one water molecule and one-half of a CdCl_6_^4-^ anion (located on a crystallographic inversion centre) (Fig. 1). The atomic arrangement of (C_11_H_18_N_2_)_2_CdCl_6_.2H_2_O can be described as built up by inorganic chains of CdCl_6_ octahedra and water molecules extending along the b direction held together by O—H···Cl hydrogen bonds (Fig. 2, Table 1). Two such chains cross the unit cell at *z* = 0, *z* = 1/2 and *x* = 1/2 (Fig. 3). The organic groups are located between these chains and connect to them through N—H···Cl, C—H···Cl and N—H···O hydrogen bonds to form a three dimensional infinite network (Fig. 3, Table 1). All the chloride ions are involved in hydrogen bonding. It should be pointed out at this point that the C—H···Cl hydrogen bonds do usually not play a large role in stabilizing a structure (Janiak & Scharmann, 2003), but due to the large number of these interactions in the title compound they seem to substantially contribute to the choice of packing observed in the structure of the title compound. Among all the hydrogen bonds, two are bifurcated: N2—H2A···(Cl1, Cl2) and N2—H2B···(Cl2, Cl3). The H1 hydrogen atom attached to the N1 nitrogen atom is bonded only to the water molecule, *via* the N1—H1···O1 hydrogen bond, and not to the CdCl_6_^4-^ anion.

The Cd II ion is in an octahedral coordination environment composed of six chloride anions as to form an hexachlorocadmate (II) ion. In this kind of anion, the Cd—Cl bond lengths and Cl—Cd—Cl bond angles are generally not equal to one another but vary with the environment around the Cl atoms (Bala *et al.*, 2006). In the title compound, the values of the Cd—Cl bond lengths vary between 2.5528 (5) and 2.7055 (4) Å. The Cl—Cd—Cl angles range from 87.354 (11) to 92.646 (11)°. These geometrical parameters agree with those found in [Co(NH_3_)_6_]_4_ [CdCl_6_] [CdCl_4_(SCN)(H_2_O)]_2_Cl_2_.2H_2_O where the Cd—Cl distances are between 2.5937 (9) and 2.691 (1) Å and the Cl—Cd—Cl angles ranging from 89.23 (3) to 95.50 (3)° (Bala *et al.*, 2006). Owing to the obvious differences of Cd—Cl distances and Cl—Cd—Cl angles in (C_11_H_18_N_2_)_2_CdCl_6_.2H_2_O, the coordination geometry of the Cd atom could be regarded as a slightly distorted octahedron which is in full agreement with the literature data (Bala, *et al.*, 2006).

### Experimental

1-Benzypyperazine (2 mmol, 0.352 g) and CdCl_2_ (1 mmol, 0.183 g), were dissolved in dilute HCl (10 ml, 1 *M*) and the resultant solution was slowly evaporated at room temperature. A crystal of the title compound, which remained stable under normal conditions of temperature and humidity, was isolated after several days and subjected to X-ray diffraction analysis (yield 55%).

### Refinement

C—H and NH_2_^+^ hydrogen atoms were placed in calculated positions with C—H in the range 0.93–0.97 and N—H equal to 0.92 Å. The N—H^+^ and the water hydrogen atom postitions were refined with N—H and O—H distance restraints of 0.91 (2) and 0.84 (2) Å. The *U*_iso_(H) values of all H atoms were constrained to 1.2 or 1.5 times *U*_eq_ of the respective parent atom.

### Crystal data

**Table:**

(C_11_H_18_N_2_)_2_[CdCl_6_]·2H_2_O	*F*(000) = 732
*M**_r_* = 717.68	*D*_x_ = 1.587 Mg m^−^^3^
Monoclinic, *P*2_1_/*c*	Mo *K*α radiation, λ = 0.71073 Å
Hall symbol: -P 2ybc	Cell parameters from 2788 reflections
*a* = 12.734 (2) Å	θ = 2.7–31.0°
*b* = 9.1686 (14) Å	µ = 1.29 mm^−^^1^
*c* = 13.216 (2) Å	*T* = 100 K
β = 103.249 (3)°	Plate, colourless
*V* = 1502.0 (4) Å^3^	0.55 × 0.45 × 0.25 mm
*Z* = 2	

### Data collection

**Table:**

Bruker SMART APEX CCD diffractometer	4446 independent reflections
Radiation source: fine-focus sealed tube	4123 reflections with *I* > 2σ(*I*)
graphite	*R*_int_ = 0.016
ω scans	θ_max_ = 31.3°, θ_min_ = 1.6°
Absorption correction: multi-scan (*SADABS*; Bruker, 2009)	*h* = −17→18
*T*_min_ = 0.622, *T*_max_ = 0.746	*k* = −12→13
11244 measured reflections	*l* = −18→19

### Refinement

**Table:**

Refinement on *F*^2^	Primary atom site location: structure-invariant direct methods
Least-squares matrix: full	Secondary atom site location: difference Fourier map
*R*[*F*^2^ > 2σ(*F*^2^)] = 0.022	Hydrogen site location: inferred from neighbouring sites
*wR*(*F*^2^) = 0.057	H atoms treated by a mixture of independent and constrained refinement
*S* = 1.07	*w* = 1/[σ^2^(*F*_o_^2^) + (0.0269*P*)^2^ + 0.6306*P*] where *P* = (*F*_o_^2^ + 2*F*_c_^2^)/3
4446 reflections	(Δ/σ)_max_ = 0.001
172 parameters	Δρ_max_ = 0.51 e Å^−^^3^
3 restraints	Δρ_min_ = −0.83 e Å^−^^3^

### Special details

**Table:**

Geometry. All e.s.d.'s (except the e.s.d. in the dihedral angle between two l.s. planes) are estimated using the full covariance matrix. The cell e.s.d.'s are taken into account individually in the estimation of e.s.d.'s in distances, angles and torsion angles; correlations between e.s.d.'s in cell parameters are only used when they are defined by crystal symmetry. An approximate (isotropic) treatment of cell e.s.d.'s is used for estimating e.s.d.'s involving l.s. planes.
Refinement. Refinement of *F*^2^ against ALL reflections. The weighted *R*-factor *wR* and goodness of fit *S* are based on *F*^2^, conventional *R*-factors *R* are based on *F*, with *F* set to zero for negative *F*^2^. The threshold expression of *F*^2^ > σ(*F*^2^) is used only for calculating *R*-factors(gt) *etc*. and is not relevant to the choice of reflections for refinement. *R*-factors based on *F*^2^ are statistically about twice as large as those based on *F*, and *R*- factors based on ALL data will be even larger.

### Fractional atomic coordinates and isotropic or equivalent isotropic displacement parameters (Å^2^)

**Table:**

	*x*	*y*	*z*	*U*_iso_*/*U*_eq_	
C1	0.03492 (12)	0.81561 (17)	0.11848 (12)	0.0242 (3)	
H1C	0.0759	0.7794	0.0722	0.029*	
C2	−0.07139 (13)	0.85867 (19)	0.08024 (14)	0.0312 (3)	
H2	−0.1032	0.8503	0.0081	0.037*	
C3	−0.13120 (12)	0.91374 (16)	0.14687 (15)	0.0292 (3)	
H3	−0.2032	0.9457	0.1202	0.035*	
C4	−0.08597 (12)	0.92209 (16)	0.25191 (14)	0.0269 (3)	
H4	−0.1272	0.9588	0.2978	0.032*	
C5	0.01972 (11)	0.87702 (15)	0.29110 (12)	0.0213 (3)	
H5	0.0500	0.8814	0.3637	0.026*	
C6	0.08142 (10)	0.82535 (13)	0.22417 (11)	0.0164 (2)	
C7	0.19878 (10)	0.78999 (13)	0.26364 (11)	0.0156 (2)	
H7A	0.2267	0.7431	0.2076	0.019*	
H7B	0.2074	0.7203	0.3222	0.019*	
C8	0.23911 (10)	1.04936 (12)	0.22283 (10)	0.0125 (2)	
H8A	0.1609	1.0712	0.2067	0.015*	
H8B	0.2591	1.0205	0.1575	0.015*	
C9	0.30187 (10)	1.18282 (12)	0.26720 (10)	0.0122 (2)	
H9A	0.2858	1.2635	0.2162	0.015*	
H9B	0.2795	1.2138	0.3309	0.015*	
C10	0.44650 (10)	1.02741 (13)	0.36618 (10)	0.0120 (2)	
H10A	0.4307	1.0540	0.4337	0.014*	
H10B	0.5244	1.0051	0.3782	0.014*	
C11	0.38168 (9)	0.89435 (12)	0.32287 (10)	0.0116 (2)	
H11A	0.4022	0.8627	0.2584	0.014*	
H11B	0.3981	0.8137	0.3738	0.014*	
Cd1	0.5000	0.5000	0.5000	0.01071 (4)	
Cl1	0.36321 (2)	0.69672 (3)	0.55240 (2)	0.01362 (6)	
Cl2	0.59831 (3)	0.49308 (3)	0.69137 (2)	0.01174 (6)	
Cl3	0.37573 (2)	0.28368 (3)	0.54229 (2)	0.01263 (6)	
N1	0.26320 (8)	0.92695 (11)	0.29971 (8)	0.01118 (18)	
N2	0.41977 (8)	1.15258 (10)	0.29270 (8)	0.01172 (19)	
H2A	0.4565	1.2342	0.3221	0.014*	
H2B	0.4416	1.1317	0.2326	0.014*	
O1	0.23289 (9)	0.99157 (10)	0.49769 (9)	0.0180 (2)	
H1	0.2483 (14)	0.9558 (19)	0.3591 (12)	0.018 (4)*	
H1A	0.2690 (17)	0.924 (2)	0.5315 (17)	0.044 (6)*	
H1B	0.2655 (18)	1.066 (2)	0.5229 (18)	0.047 (7)*	

### Atomic displacement parameters (Å^2^)

**Table:**

	*U*^11^	*U*^22^	*U*^33^	*U*^12^	*U*^13^	*U*^23^
C1	0.0181 (7)	0.0284 (7)	0.0253 (7)	−0.0036 (5)	0.0031 (6)	−0.0025 (6)
C2	0.0203 (7)	0.0358 (8)	0.0323 (9)	−0.0051 (6)	−0.0049 (6)	0.0040 (7)
C3	0.0131 (6)	0.0210 (6)	0.0504 (10)	−0.0028 (5)	0.0008 (6)	0.0050 (6)
C4	0.0169 (7)	0.0201 (6)	0.0458 (10)	−0.0028 (5)	0.0117 (6)	−0.0048 (6)
C5	0.0166 (6)	0.0208 (6)	0.0274 (7)	−0.0038 (5)	0.0068 (5)	−0.0034 (5)
C6	0.0113 (6)	0.0134 (5)	0.0241 (7)	−0.0034 (4)	0.0033 (5)	−0.0013 (5)
C7	0.0132 (6)	0.0106 (5)	0.0223 (6)	−0.0020 (4)	0.0027 (5)	−0.0012 (4)
C8	0.0126 (5)	0.0112 (5)	0.0129 (6)	0.0007 (4)	0.0013 (4)	0.0025 (4)
C9	0.0122 (5)	0.0106 (5)	0.0144 (5)	0.0007 (4)	0.0042 (4)	0.0005 (4)
C10	0.0114 (5)	0.0129 (5)	0.0111 (6)	0.0004 (4)	0.0014 (4)	0.0004 (4)
C11	0.0096 (5)	0.0116 (5)	0.0136 (5)	0.0017 (4)	0.0028 (4)	0.0007 (4)
Cd1	0.01320 (7)	0.00972 (6)	0.00955 (7)	−0.00012 (4)	0.00332 (5)	0.00002 (4)
Cl1	0.01548 (14)	0.01289 (12)	0.01327 (13)	0.00202 (10)	0.00489 (11)	0.00058 (10)
Cl2	0.01277 (14)	0.01112 (12)	0.01138 (14)	−0.00071 (8)	0.00289 (11)	0.00010 (9)
Cl3	0.01428 (13)	0.01192 (12)	0.01209 (13)	−0.00170 (9)	0.00382 (10)	−0.00078 (9)
N1	0.0112 (5)	0.0100 (4)	0.0127 (5)	−0.0004 (3)	0.0032 (4)	0.0003 (4)
N2	0.0119 (5)	0.0110 (4)	0.0131 (5)	−0.0008 (3)	0.0046 (4)	−0.0005 (4)
O1	0.0185 (5)	0.0165 (4)	0.0190 (5)	0.0001 (3)	0.0046 (4)	−0.0001 (3)

### Geometric parameters (Å, °)

**Table:**

C1—C2	1.389 (2)	C9—H9A	0.9900
C1—C6	1.390 (2)	C9—H9B	0.9900
C1—H1C	0.9500	C10—N2	1.4915 (16)
C2—C3	1.385 (3)	C10—C11	1.5102 (17)
C2—H2	0.9500	C10—H10A	0.9900
C3—C4	1.378 (3)	C10—H10B	0.9900
C3—H3	0.9500	C11—N1	1.4992 (15)
C4—C5	1.390 (2)	C11—H11A	0.9900
C4—H4	0.9500	C11—H11B	0.9900
C5—C6	1.3938 (19)	Cd1—Cl2^i^	2.5528 (5)
C5—H5	0.9500	Cd1—Cl2	2.5528 (5)
C6—C7	1.5012 (18)	Cd1—Cl3	2.6751 (4)
C7—N1	1.5158 (15)	Cd1—Cl3^i^	2.6751 (4)
C7—H7A	0.9900	Cd1—Cl1^i^	2.7055 (4)
C7—H7B	0.9900	Cd1—Cl1	2.7055 (4)
C8—N1	1.4978 (15)	N1—H1	0.889 (14)
C8—C9	1.5051 (16)	N2—H2A	0.9200
C8—H8A	0.9900	N2—H2B	0.9200
C8—H8B	0.9900	O1—H1A	0.837 (16)
C9—N2	1.4875 (15)	O1—H1B	0.830 (16)
			
C2—C1—C6	120.17 (15)	C11—C10—H10A	109.5
C2—C1—H1C	119.9	N2—C10—H10B	109.5
C6—C1—H1C	119.9	C11—C10—H10B	109.5
C3—C2—C1	120.28 (16)	H10A—C10—H10B	108.1
C3—C2—H2	119.9	N1—C11—C10	110.70 (9)
C1—C2—H2	119.9	N1—C11—H11A	109.5
C4—C3—C2	119.81 (14)	C10—C11—H11A	109.5
C4—C3—H3	120.1	N1—C11—H11B	109.5
C2—C3—H3	120.1	C10—C11—H11B	109.5
C3—C4—C5	120.30 (15)	H11A—C11—H11B	108.1
C3—C4—H4	119.8	Cl2^i^—Cd1—Cl2	180.0
C5—C4—H4	119.8	Cl2^i^—Cd1—Cl3	92.646 (11)
C4—C5—C6	120.22 (14)	Cl2—Cd1—Cl3	87.354 (11)
C4—C5—H5	119.9	Cl2^i^—Cd1—Cl3^i^	87.354 (11)
C6—C5—H5	119.9	Cl2—Cd1—Cl3^i^	92.646 (11)
C1—C6—C5	119.18 (13)	Cl3—Cd1—Cl3^i^	180.0
C1—C6—C7	119.76 (12)	Cl2^i^—Cd1—Cl1^i^	87.784 (12)
C5—C6—C7	120.93 (13)	Cl2—Cd1—Cl1^i^	92.218 (12)
C6—C7—N1	110.75 (10)	Cl3—Cd1—Cl1^i^	90.322 (15)
C6—C7—H7A	109.5	Cl3^i^—Cd1—Cl1^i^	89.680 (15)
N1—C7—H7A	109.5	Cl2^i^—Cd1—Cl1	92.215 (12)
C6—C7—H7B	109.5	Cl2—Cd1—Cl1	87.783 (11)
N1—C7—H7B	109.5	Cl3—Cd1—Cl1	89.678 (14)
H7A—C7—H7B	108.1	Cl3^i^—Cd1—Cl1	90.321 (14)
N1—C8—C9	109.69 (10)	Cl1^i^—Cd1—Cl1	180.0
N1—C8—H8A	109.7	C8—N1—C11	109.14 (9)
C9—C8—H8A	109.7	C8—N1—C7	113.30 (10)
N1—C8—H8B	109.7	C11—N1—C7	110.23 (9)
C9—C8—H8B	109.7	C8—N1—H1	108.9 (11)
H8A—C8—H8B	108.2	C11—N1—H1	106.7 (11)
N2—C9—C8	110.79 (9)	C7—N1—H1	108.3 (12)
N2—C9—H9A	109.5	C9—N2—C10	111.02 (9)
C8—C9—H9A	109.5	C9—N2—H2A	109.4
N2—C9—H9B	109.5	C10—N2—H2A	109.4
C8—C9—H9B	109.5	C9—N2—H2B	109.4
H9A—C9—H9B	108.1	C10—N2—H2B	109.4
N2—C10—C11	110.58 (10)	H2A—N2—H2B	108.0
N2—C10—H10A	109.5	H1A—O1—H1B	103 (3)
			
C6—C1—C2—C3	1.0 (2)	N1—C8—C9—N2	59.09 (13)
C1—C2—C3—C4	−1.8 (2)	N2—C10—C11—N1	−56.82 (13)
C2—C3—C4—C5	0.7 (2)	C9—C8—N1—C11	−59.78 (12)
C3—C4—C5—C6	1.1 (2)	C9—C8—N1—C7	176.99 (10)
C2—C1—C6—C5	0.8 (2)	C10—C11—N1—C8	59.02 (13)
C2—C1—C6—C7	−175.11 (13)	C10—C11—N1—C7	−175.95 (10)
C4—C5—C6—C1	−1.8 (2)	C6—C7—N1—C8	−48.38 (14)
C4—C5—C6—C7	173.99 (12)	C6—C7—N1—C11	−171.01 (10)
C1—C6—C7—N1	109.33 (14)	C8—C9—N2—C10	−56.81 (13)
C5—C6—C7—N1	−66.48 (15)	C11—C10—N2—C9	55.34 (12)

Symmetry codes: (i) −*x*+1, −*y*+1, −*z*+1.

### Hydrogen-bond geometry (Å, °)

**Table:**

*D*—H···*A*	*D*—H	H···*A*	*D*···*A*	*D*—H···*A*
N2—H2A···Cl1^ii^	0.92	2.58	3.3383 (11)	140
N2—H2A···Cl2^ii^	0.92	2.59	3.2672 (11)	131
N2—H2B···Cl2^iii^	0.92	2.47	3.1846 (11)	135
N2—H2B···Cl3^iii^	0.92	2.58	3.2799 (12)	133
N1—H1···O1	0.89 (1)	1.92 (1)	2.7945 (16)	170 (2)
O1—H1A···Cl1	0.84 (2)	2.39 (2)	3.1678 (11)	155 (2)
O1—H1B···Cl3^iv^	0.83 (2)	2.42 (2)	3.2152 (11)	161 (2)
C9—H9A···Cl3^iii^	0.99	2.83	3.331 (2)	112
C9—H9B···Cl3^iv^	0.99	2.85	3.659 (3)	139
C10—H10A···Cl3^iv^	0.99	2.73	3.565 (2)	143
C10—H10B···Cl2^iii^	0.99	2.84	3.340 (4)	112
C11—H11A···Cl1^iii^	0.99	2.71	3.626 (1)	154
C11—H11B···Cl1	0.99	2.72	3.587 (1)	146

Symmetry codes: (ii) −*x*+1, −*y*+2, −*z*+1; (iii) *x*, −*y*+3/2, *z*−1/2; (iv) *x*, *y*+1, *z*.
